# The association between probationers’ addiction levels and socioeconomic-psychological characteristics

**DOI:** 10.1186/s40359-023-01142-1

**Published:** 2023-04-06

**Authors:** Inci Derya Yucel, Gorkem Yararbas

**Affiliations:** Institute on Drug Abuse, Toxicology and Pharmaceutical Science, Ege University, Bornova/İzmir, Turkey

**Keywords:** Addiction, Probation, Rehabilitation, Socioeconomic-psychological characteristics

## Abstract

**Objective:**

The aim of this study is to evaluate probationers’ addiction levels and associated socioeconomic and psychological features in Izmir Probation Directorate.

**Methods:**

This cross-sectional study was conducted in Izmir Probation Directorate between August 27, 2018 and November 27, 2018. The study’s dependent variable was adult probationers’ addiction level which was evaluated by the Addiction Profile Index-Clinical Version (API-C). The independent variables were individual factors, social environmental factors, API-C scale subdimensions and perceived social support. For paired comparisons, Student’s t test and ANOVA were used. Linear regression analysis was used for multiple comparisons. p < 0.05 was accepted as the limit of statistical significance.

**Results:**

A total of 200 male probationers participated in the study (82.3%, n = 243). The participants’ average age was 29.9 ± 7.7. The participants’ average addiction score was 5.65 ± 2.33. According to the results of the regression analysis; education level (B = 1.438, 95% CI 0.936, 1.941, *p* < 0.001) working status at a job (B = 2.687, 95% CI 1.428, 3.945, *p* < 0.001) father’s education level (B=-1.117, 95% CI -1.473, -0.762, *p* < 0.001) and anger management problems (B = 0.750, 95%CI 0.517, 0.982, *p* < 0.001) were explanatory for addiction level. The model was 50.8% explanatory of addiction level (p < 0.05).

**Conclusion:**

Probationers who grew up with only their mother, whose father had a low education level, who had higher levels of anger management problems and who were unemployed had higher levels of addiction. These results emphasize the need for social work in the rehabilitation processes of individuals. Treating the risk factors indicated by the study results as screening and follow-up parameters in the probation population can be useful in improving the success of the probation program.

## Introduction

It is observed that the prevalence of substance use and detection in adults has increased in Turkey and throughout Europe [1]. Rehabilitation facilities differ in their contents and process management, national legislation, culture and other variables which include needs and regulations in probation around the world.

In Turkey, the probation system was established in 2005 by the Republic of Turkey Ministry of Justice General Directorate and Detention Houses with the aim of reintegrating criminal adults and children who have difficulties with the law in terms of execution and rehabilitation models. The scope of probation has been extended, and probation directorates’ workload and responsibilities have increased dramatically. According to Article 191 of the Turkish Penal Code (TPC) No 5237, a person who buys, accepts or holds drugs or stimulants for use or who uses drugs or stimulants is given a probation sentence with/without treatment that lasts for at least 1 year and that includes executions, training and rehabilitation. Substance user probationers have responsibilities and obligations under probation supervision such as individual interviews, attending the “Cigarette, Alcohol and Substance Addiction Programme-Probation Version (SAMBA-DS)” [2] and seminars.

Addiction treatment, as in all addiction types, consists of several cognitive, behavioral and emotional stages. These stages are defined as precontemplation, contemplation, preparation, action, and maintenance according to the Transtheoretical Model [3]. Related to these stages, probation directorates provide support to probationers to renew and improve living standards to change their lives and ensure that they stay away from substance use [4].Probation is associated with holding or using substance. A small proportion of probationers show severe symptoms of addiction and whole group average addiction scores are relatively low compared to the patients in substance addiction treatment centers (AMATEM) [5]. The overall success rates of probation programs are limited and repeated substance use is common in this population. High levels of repeated substance use in a relatively low addicted group indicate the need for a specific approach for this special group. Therefore, psychological and social determinants of probationers’ addiction level should be further investigated. In order to keep individuals away from substance use, there is a need for new approaches that support the ability to adapt to life, such as anger management, that allow education, profession and job opportunities instead of programs built only on medical treatment perspective. This research may inspire other authorities in different countries that aim to improve probation programs.

Although probation is based on the monitoring of substance use for legal reasons, individuals also need to be closely monitored in terms of psychological and social parameters due to the complex nature of addiction. Although there are studies examining the socioeconomic and psychological characteristics of probationers around the world [6–9], it is necessary to examine the patterns of different populations. Examination and a better understanding of addiction levels and related socioeconomic and psychological factors will provide the opportunity to bring a new perspective and approach to different probation programs used worldwide.

The aim of this study is to evaluate probationers’ addiction levels and associated socioeconomic and psychological features in Izmir Probation Directorate. These features are age, marital status, education level, working status, criminal background, father’s education level, having a caregiver during childhood for socioeconomic factors and anger management problems, anxiety, lack of assertiveness, depression, impulsivity, novelty-seeking behavior, and perceived social support for psychological factors.

## Methods

The cross-sectional study was conducted in Izmir Probation Directorate in the province of Izmir. Izmir Probation Directorate is one of the largest probation directorates in Turkey, serving the majority of the districts in the province of Izmir.

It was planned to include all participants (n = 243) who applied to Izmir Probation Directorate during the 3-month period between August 27 and November 27, 2018. 21 of them registered for probation but did not participate in the probation program; 15 participants did not complete the data collection forms adequately and 7 participants did not volunteer to participate in the study. Inclusion criteria were being 18 years of age or older, being male, not having a severe psychopathological disease (such as schizophrenia), participating in and completing individual interviews, participating in group work intervention programs in the probation directorate and agreeing to take part in the study. The participants completed the scales and questionnaire through self-reporting.

The present study’s dependent variable was adult probationers’ addiction level. The addiction level was evaluated by the Addiction Profile Index-Clinical Version (API-C) developed by Ogel et al. [5]. The API-C scale was applied to the participants to obtain information about their current substance use characteristics and mental and personal status of substance use during the probation process. The API-C includes the evaluation of 6 dimensions that continue and accompany addiction, apart from the dimensions directly related to addiction. These are depression, anxiety, anger control failure, lack of safe behavior, novelty-seeking behavior and impulsivity. Two of these measure mental status and others measure the personal characteristics related to addiction. The scale consists of 58 items. Below 12 points indicates a low addiction level, between 12 and 14 points indicates a medium addiction level, and above 14 points indicates a high addiction level.

The study’s independent variables were age, marital status, level of education, working status at a job, and criminal background in the scope of individual factors; father’s education level and having a caregiver during childhood in the scope of the social environmental factors; anger management problems, anxiety, lack of assertiveness, depression, impulsivity, and novelty seeking behavior in the scope of the API-C Scale subdimensions; and perceived social support.

The independent variables of the individual and social environmental factors were collected by the “Case Information Form” developed by the research team and it determines probationers’ demographic features which can be relevant to substance abuse/addiction behaviors and criminal background.

Perceived social support was collected by “The Multidimensional Scale of Perceived Social Support (MSPSS), which was developed by Zimet et al. (1990) [10] and measures the adequacy of social support from three different dimensions with 12 items: family, friends and a special person. Each item was graded using an individual 7-interval scale [11].

IBM SPSS Statistics for Windows, version 25.0 [12] was used for linear regression analysis. For paired comparisons, Student’s t test and ANOVA were used while opting for multiple linear regression analysis in multivariate comparisons. Factors that were revealed to be associated with addiction levels in univariate analyses were then examined by multilevel analysis. P < 0.05 was accepted as the limit of statistical significance for all analysis. We constructed models using associated factors and examined to what extent they were explanatory of addiction level.

This research was carried out with the approval of the Research Ethics Committee of the Medical Faculty of Ege University (decision n. 05/06/2018-18-6/37). This research; with the application permit approval of the TR Ministry of Justice, General Directorate of Prisons and Detention Houses dated July 17, 2018 and numbered 46,985,942/679/10,291, was carried out in the individual meeting room at Izmir Probation Directorate. Informed consent was obtained from all participants. This study followed the Declaration of Helsinki.

## Results

In this study, subareas of substance use disorder, such as substance use characteristics (type of substance used and frequency of use), diagnostic criteria, effects of substance use on the life of the individual, craving and motivation to quit the substance, and related psychological factors, were examined.

**Fig. 1:**
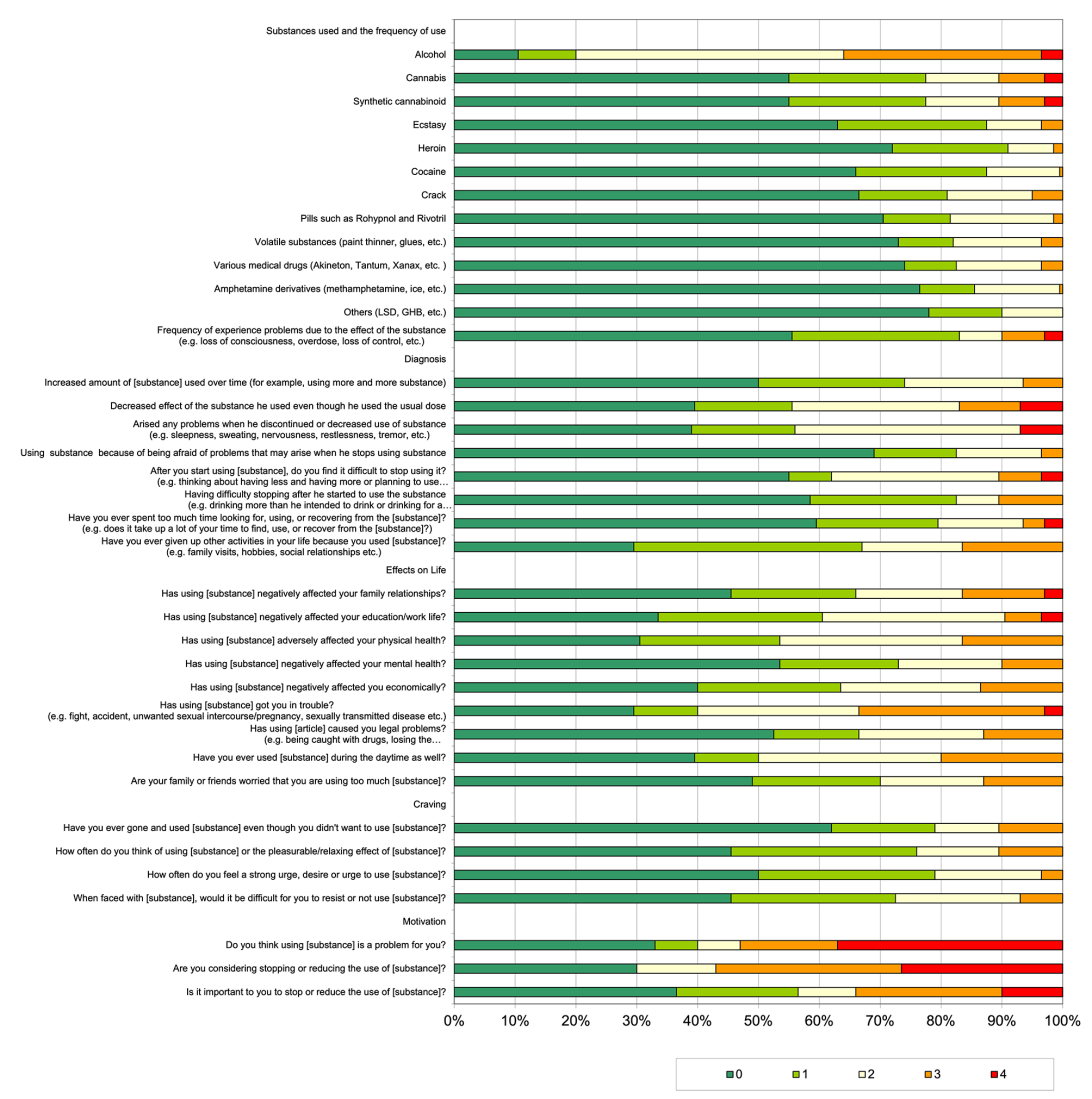
Sub-areas which are related to substance use disorder due to API-C: *For items 1–11; ‘0’ Never, ‘1’ Only once or twice, ‘2’ 1–3 times a month, ‘3’ 1–5 times a week, ‘4’ Almost every day

The distribution of the subareas related to substance use disorders including substance use and frequency of substance use, diagnosis, effects on life, craving and motivation due to the Addiction Profile Index-Clinical Form is presented in Fig. 1. The participants had more background about using alcohol (89.5%), cannabis (45%) and synthetic cannabinoids (45%)than other substances (between 22% and 37%) at least once or twice in a week. Among the participants, 17% stated that they often or almost always had symptoms such as sleepiness, sweating, nervousness, restlessness, and tremors. A total of 10.5% of the participants said that after they started using substances, they found it difficult to stop using (e.g., thinking about having less and having more or planning to use it for a short time and using it for a long time). 33% of the participants stated that they gave up other activities in their lives since they used substances (e.g., family visits, hobbies, social relationships, etc.). A total of 33.5% of them clarified that using substances had gotten them in trouble (e.g., fights, accidents, unwanted sexual intercourse/pregnancy, sexually transmitted diseases). 50% of them used substances during the daytime as well. Among the participants, 49% clarified that their families or friends did not worry since the participants used substances too much. 33% of the participants thought that using substances was not a problem for them at any time. 57% of the participants reported that they almost always or often considered stopping or reducing the use of substances, while 34% of them thought that it was almost always or often important for them to stop or reduce the use of substances (Fig. 1).

**Fig. 2:**
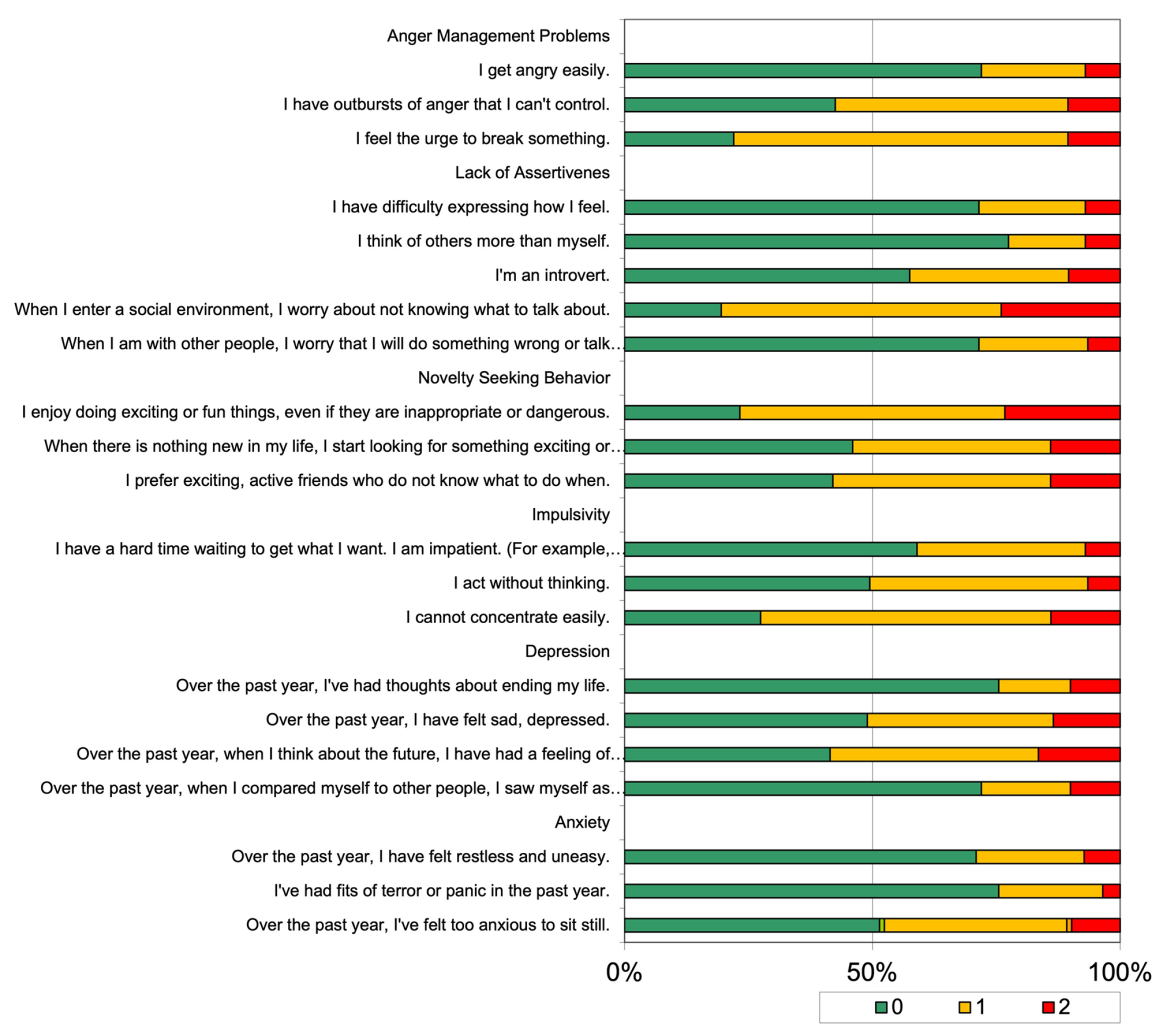
6 sub-areas including anger management problems, lack of assertiveness, novelty seeking behavior, impulsivity, depression, anxiety which are related to mental health and traits due to API-C:*For items ‘0’ Never, ‘1’ Sometimes, ‘2’ Almost always

Figure 2 shows the distribution of the 6 subareas, including anger management problems, lack of assertiveness, novelty-seeking behavior, impulsivity, depression, and anxiety, according to the API-C.

A total of 10.5% of the participants clarified that they always had outbursts of anger that they could not control. 24% of them reported that when they entered a social environment, they always worried about not knowing what to talk about. Among the participants 14% stated that when there was nothing new in their lives, they always started looking for something exciting or exhilarating. A total of 16.5% of the participants clarified that over the past year, when they thought about the future, they always had a feeling of hopelessness. Among the participants 10% stated that over the past year, they always had felt too anxious to sit still (Fig. 2).

Two hundred male probationers participated in the study (82.3%, n = 243). The participants’ average age was 29.9 ± 7.7. Individual factors, social environment and API-C subdimension variables associated with the addiction level of participants are shown in Table 1. 86% of the participants were aged 35 and under, 67% were single, 63.3% had a high school diploma or higher, 89.5% had a job, and 79.5% had no criminal history (Table 1). The participants’ Addiction Profile Index-Clinical Form (API-C) average score was 5.65 ± 2.33. In this study, it was found that all participants had low addiction severity.

**Table 1:** Individual and Social Environmental Factors Associated with Addiction Profile and Perceived Social Support

	Distribution of Participants		Addiction Level		p
	n	%	API-C-API Score (Mean)	±SD	
Individual Factors					
Age					
35 and under	172	86	3.85	± 2.83	0.002*
35+	28	14	3.94	± 2.85	
Marital Status					
Married	66	33	4.51	± 3.04	0.001*
Single	134	67	6.11	± 2.81	
Education Level					
Primary School	21	10.5	5.31	± 2.78	< 0.001**
Middle School	52	26	3.58	± 2.44	
High School and above	127	63.5	6.44	± 2.82	
Working Status at a Job					
Yes	179	89.5	5.15	± 2.78	< 0.001**
No	21	10.5	9.23	± 1.94	
Criminal Background					
Yes	41	20.5	5.15	± 2.78	0.027*
No	159	79.5	9.23	± 1.94	
Social Environmental Factors					
Father-Education Level					
Primary School	34	17	7.40	± 1.10	< 0.001**
Middle School	105	52.5	5.18	± 3.14	
High School and Above	61	30.5	5.35	± 3.01	
Caregiver for Growing up in Childhood					
Mother and Father	179	89.5	5.58	± 3.05	0.001*
Only Mother	7	3.5	8.25	± 0.25	
Relatives	7	3.5	5.64	± 0.00	
Dormitory/Institution	7	3.5	2.87	± 0.26	
API-C Sub-Dimensions					
Anger Management Problems					
Yes	103	51.5	7.10	± 2.41	< 0.001**
No	97	48.5	3.97	± 2.66	
Anxiety					
Yes	63	31.5	6.65	± 3.11	0.001**
No	137	68.5	5.09	± 2.79	
Lack of Assertiveness					
Yes	43	21.5	4.08	± 2.48	< 0.001**
No	157	78.5	5.17	± 2.73	
Depression					
Yes	56	28	6.94	± 2.89	< 0.001**
No	144	72	5.05	± 2.85	
Impulsivity					
	200	100	0.273***		< 0.001**
Novelty Seeking Behavior					
	200	100	0.487***		< 0.001**
Perceived Social Support					
Family			-0.10		0.16
Friends	200	100	0.01		0.94
A Special Person			-0.08		0.28
Total Score	200	100	-0.07		0.32

Note: *p < 0.05, **p < 0.001, API-C-API: Addiction Profile Index-Clinical Form Addiction Level, SD: Standard Deviation, *** Correlation Coefficient

The addiction level score of the participants who were aged 35 and under, who were single, who had a higher education level, who did not have a job, who did not have a criminal background, whose father’s education level was lower, who were raised by only their mothers, who had more anger management problems and anxiety, who were more assertive and more depressive was significantly higher (p < 0.05). The participants with higher impulsivity and novelty seeking behavior scores had significantly higher addiction levels (p < 0.001) (Table 1).

Table 2 shows the results of the multiple linear regression analysis which were adjusted for all variables identified to be associated with addiction level. According to the regression analysis, education level (B: 1.438, 95%CI: 0.936–1.941), working status at a job (B: 2.687, 95%Cl: 1.428–3.945), father’s education level (B: -1.117, 95%Cl: -1.473– -0.762), and anger management problems (B: 0.750, 95%Cl: 0.517–0.982) were explanatory of addiction level. The model was 50.8% explanatory of the addiction level. Although the level of addiction is mainly associated with the reinforcing pharmacological effects of substances, the results of the present study reveal the importance of socioeconomic and psychological parameters such as anger management.

**Table 2:** Multiple Linear Regression Analysis of API-C Score Determinants

	Model			
	Unstandardized Coefficient			
Individual Factors	**B**	**95% CI**		
Age (35 and under/35+)	-0.132	-0.459-0.196		
Marital Status (Married/Single)	-0.679	-1.420-0.062		
Education Level (Primary/Secondary/High School/ Associate Degree)	1.438	0.936–1.941**		
Working Status at a Job (Yes/No)	2.687	1.428–3.945**		
Criminal History (Yes/No)	0.713	-0.102-1.529		
Social Environmental Factors				
Father-Education Level (Primary/Middle/High School/Associate/Undergraduate)	-1.117	-1.473 - -0.762**		
Caregiver for growing up (Mother and Father/Only Mother/Relatives/Dormitory, Institution)	0.027	-0.217-0.270		
API-C-Sub Dimensions				
Anger Management Problems (Yes/No)	0.750	0.517–0.982**		
Perceived Social Support				
Family	-0.049	-0.133-0.034		
Friend	0.045	-0.040-0.129		
A special person	-0.035	-0.116-0.045		
Total Score	-0.014	-0.040-0.013		
Explanatory and significance levels of the models				
R^2^:	0.508			
Adj. R^2^:	0.488			
p	<0.001**			

Note: R²: Coefficient of determination; *p < 0.05; **p < 0.001. CI: Confidence Interval. The analysis was adjusted for all variables

## Discussion

This research was carried out in Izmir Probation Directorate to determine the relationship between addiction levels and sociodemographic and psychological characteristics of 200 adult individuals who have substance use problems.

In our study, it was determined that the individuals who were over 35 years old, single, had a high school level of education level and above, were unemployed, had no criminal history, whose fathers had a low level of education and were raised by only their mother showed significantly higher levels of addiction. In addition, it was found that individuals with high levels of anger, anxiety, depression, impulsiveness, novelty, and lack of assertiveness also had significantly higher levels of addiction. In the results of multivariate analysis, having a high school level of education level and above, being unemployed, whose father’s level of education was low and who had high levels of anger and control problems remained significant in the model.

In the present study, the participants’ addiction score was 5.65 ± 2.33, which refers to a low addiction level according to the API-C which has similar scores to a previous study with similar sample [2]. However, the patients in substance addiction treatment centers (AMATEM) had higher addiction levels [5] which suggest that probationers with substance use problems differ from the patients in AMATEM due to their low level of addiction. This difference points to the need for a unique approach to the probation group.

In this study, the addiction scores of participants with high school education and above were higher. Many studies show that higher education levels are associated with higher rates of alcohol and marijuana use [13, 14]. This relationship is also influenced by many factors, such as school type and duration of education [15]. Rarely there are studies which show no relationship between addiction and education level [16]. While there is no consensus on the relationship between socioeconomic variables such as income and wealth and addiction level [17], the fact that substances are more available for highly educated people may be a risk factor. The addiction scores of the unemployed participants were found to be higher in the present study. Several studies have found that there is a relationship between being unemployed and substance use [18, 19]. Due to the cross-sectional study design of the present study, the association should be discussed in two ways. It is possible that individuals with substance use problems may have unemployment problems due to their low level of education [20] and their inadequacy in their professional skills [21]. However, when the issue is viewed from the unemployment point of view, unemployment means more than a job loss [22]. Contrary to these data, in a study no correlation between the current working status and the level of addiction level was found [23]. This contradiction may be related to different characteristics of the study populations. Psychological features such as identity and self-esteem are also highly affected. This phenomenon is common in different types of addictions [24]. This study indicated that growing up without fathers was associated with high addiction scores. One of the most common problems among the fatherless (the father’s leaving home or death) children in different societies is substance use problems [25, 26]. In the absence of the father, the child’s acquaintance with substances and their continued use may be affected by economic and cultural factors. The economic burden of the family is typically borne by fathers in Turkish society, and it is common for juveniles to try to compensate for this economic loss. This situation causes early termination of education of the children and to come into contact with an uncontrolled environment at his early age. This may also be related to the mother’s low parenting skills due to the extra responsibilities that she has to take regarding the child and the family [27]. Lack of authority at home and exposure to neglect may also be associated with tendency to use substances.

In our study, a relationship between participants’ anger control problem level and substance abuse scores was determined. Due to the nature of addiction, the intoxicating effects of drugs, cravings and withdrawal may trigger the emergence of anger management problems [28]. Antisocial personality disorder is more frequent in probation groups [8] and the prevalence of substance use [29–31] is higher in individuals with antisocial personality disorder and anger control problems [32]. However, the participants in the present study may lack anger management skills regardless of substance use.

The educational status of the parents of individuals with substance use problems is generally low [17, 33, 34]. Similarly, the level of substance use disorder among the participants was found to be higher in those whose fathers had a low education level in the present study. This may be associated with poor parenting skills, which might cause a lack of a close relationship between parents and children. Therefore, this situation may have increased the risk of problematic social interactions. When the influencing factors of low-educated fathers and high levels of education are examined, the high addiction scores found may be explained by upward social mobility. In the literature, although there are studies that mainly point out the negative health outcomes of downward social mobility [35, 36], the results obtained in our study may differ depending on the social dynamics of the country and group where the study was conducted. Since the early 1990s, there has been intense internal migration mobility from east to west and from rural to urban in Turkey [37]. Addiction might be “a way of adapting to dislocation” [38]. Social dislocation refers to the loss of identity or culture in individuals and groups [39]. The frequency of addiction problems may have increased due to the inability to maintain family traditions and the difficulties they experience in integrating into the new environment they have started to live in, although the young people who migrated to the big cities have the opportunity to receive a relatively better education.

### Strengths and limitations

Although underreporting may be a possible bias in this type of research, most studies have found self-reports on addiction levels to be reliable, consistent and accurate [40, 41]. An additional confounding factor may be the influence of being under the sentence of the probation system. The reasons and results were examined simultaneously in the present study due to the cross-sectional design of the study, which may have affected the results. No female probationers were included in the study due to the difficulties in the number of women probationers. This was related to the gender distribution of probationers in Turkey, which was similar to other studies in the literature [42]. However, due to the nature of the samples in the probation system, we may assume that the number of women is negligible. The strength of the present research is the examination of individual and psychological characteristics that may affect the addiction levels as well as the determination of substance use characteristics of the probationers.

## Conclusions

Examining the addiction levels of probation obliged individuals in this study will contribute to both the development of protective measures and the structuring of rehabilitation activities carried out during the probation process according to needs. Although the probation process demonstrates positive gains regarding the substance use of individuals, it is remarkable that risk factors such as anger control problems still continue in light of the findings of this study. Additionally, probationers who grew up with only their mother, whose father had a low level of education and unemployed individuals had higher levels of addiction. These social variables point to the multidimensional nature of addiction and emphasize improvements in the field of social work in the rehabilitation processes of individuals. Treating the risk factors indicated by the study results as screening and follow-up parameters in the probation population can be useful for improving the success of the probation program.

## Data Availability

The datasets used and/or analyzed during the current study are available from the corresponding author on request.
